# Factors Associated with Self-Reported Previous HPV Testing Among Women Attending a Primary Healthcare Facility in Istanbul, Türkiye: A Cross-Sectional Study

**DOI:** 10.3390/healthcare14203428

**Published:** 2026-10-14

**Authors:** Bekir Aktura, Zehra Saltıkalp Sert, Esmehan Ayşit

**Affiliations:** 1 Department of Family Medicine, Faculty of Medicine, Atlas University, 34408 Istanbul, Türkiye; 2 Public Health Services, Health Directorate of Istanbul, 34122 Istanbul, Türkiye; 3 Sancaktepe District Health Directorate, 34785 Istanbul, Türkiye

**Keywords:** cervical cancer screening, human papillomavirus DNA tests, primary health care, health awareness, depression

## Abstract

**Highlights:**

**What are the main findings?**

- Of 441 women aged 30–65 attending a primary healthcare facility in Istanbul, 54.2% reported having previously undergone HPV testing.
- Awareness of HPV testing availability was strongly associated with previous testing; however, because both were assessed at the same interview, the direction of this association cannot be determined.

**What are the implications of the main findings?**

- Beck Depression Inventory scores were not clearly associated with previous HPV testing, whether analyzed continuously or categorically.
- Primary care visits may provide an opportunity to discuss cervical screening with women in the target age range, although this study did not evaluate an intervention.

**Abstract:**

**Background/Objectives**: This study aimed to estimate the proportion of women attending a primary healthcare facility in Istanbul, Türkiye, who reported previous human papillomavirus (HPV) testing and to examine associated factors, particularly depressive symptoms. **Methods:** This cross-sectional study included 507 women aged ≥18 years attending a Healthy Life Center in 2025. Sociodemographic characteristics, reproductive history, awareness of HPV testing availability and depressive symptoms (Beck Depression Inventory, BDI; total score 0–63) were recorded in face-to-face interviews. Two multivariable logistic regression models, without (Model A) and with (Model B) awareness, were fitted in the 441 women aged 30–65. **Results:** Of these women, 239 (54.2%) reported previous testing. In Model B, previous testing was associated with age (adjusted odds ratio [aOR] = 1.07 per year; 95% confidence interval [CI]: 1.04–1.11) and awareness (aOR = 21.35; 95% CI: 8.07–56.51). A history of pregnancy loss or curettage was also associated with previous testing, but this finding was considered exploratory. The BDI total score was not clearly associated with previous testing in either model (Model B aOR = 0.99 per point; 95% CI: 0.97–1.01). **Conclusions:** Awareness was strongly associated with previous testing, but because both were reported at the same interview, the direction of this association is unknown, and no effect of awareness-raising can be inferred. No clear association was found with depressive symptoms, although a modest association cannot be excluded. These clinic-based, self-reported data do not reflect population screening coverage.

## 1. Introduction

Cervical cancer remains a significant public health problem among women worldwide [1,2]. The degree of oncogenicity of the human papillomavirus (HPV) type, immune status, presence of other sexually transmitted infections, number of births, first pregnancy at a young age, use of hormonal contraceptives, and smoking are factors that increase the risk of developing cancer [2]. HPV infection with oncogenic HPV types 16 and 18 is the main etiological factor for cervical cancer, and the disease can be largely prevented with effective screening and vaccination programs [2,3]. To accelerate the elimination of cervical cancer, the World Health Organization (WHO) has set three targets for 2030: 90% of girls fully vaccinated against HPV by the age of 15, 70% of women screened with a high-performance test by the age of 35 and again by the age of 45, and 90% of women identified with cervical disease receiving treatment and care [2]. HPV DNA-based testing is more sensitive than cytology for detecting cervical precancer [4]. The 2025 European guidelines recommend HPV detection testing as the primary test in organized population-based screening programs for individuals aged 30–50 years [5].

In Türkiye, HPV DNA testing is offered to women aged 30–65 every five years as part of the National Cervical Cancer Screening Program [6]. The test is provided free of charge and is delivered through Family Health Centers, Cancer Early Diagnosis, Screening and Education Centers (KETEM) and Healthy Life Centers [7]. Although the program is widely available in primary care, participation is still incomplete. In the 2022 Türkiye Health Survey, 48.6% of women aged 30–65 reported that they had ever been screened for cervical cancer, and 37.2% had been screened within the recommended interval [8]. A regional evaluation concluded that more women need to be included in the program and recommended awareness activities through primary care staff [7]. Reviews have described barriers related to health literacy, knowledge, perceptions and the experience of screening itself [9,10]. A qualitative study of mothers of adolescent daughters in Korea also reported limited awareness of cervical cancer prevention and of the importance of regular screening [11].

Psychosocial factors may influence the use of preventive health services [12]. However, evidence on the relationship between depressive symptoms and cervical cancer screening remains inconclusive [13]. This uncertainty provides a rationale for examining depressive symptoms alongside other factors associated with previous HPV testing in our study. The US Preventive Services Task Force recommends screening adults for depression in primary care [14], so primary care is a suitable setting to examine whether depressive symptoms are related to lower participation in cervical screening. Few studies have examined this question in primary care settings in Türkiye. Therefore, this study aimed to estimate the proportion of women attending a Healthy Life Center in Istanbul who reported previous HPV testing and to examine the sociodemographic, clinical and psychosocial factors associated with it. A secondary aim was to examine whether depressive symptoms were associated with previous testing.

## 2. Materials and Methods

This cross-sectional study was conducted at the Bağcılar Sancaktepe Healthy Life Center in Istanbul between 2 January and 31 December 2025. Healthy Life Centers are multipurpose primary care units established by the Ministry of Health to strengthen preventive services and improve access to them [15]. The study is reported in accordance with STROBE guidelines.

**Figure 1:**
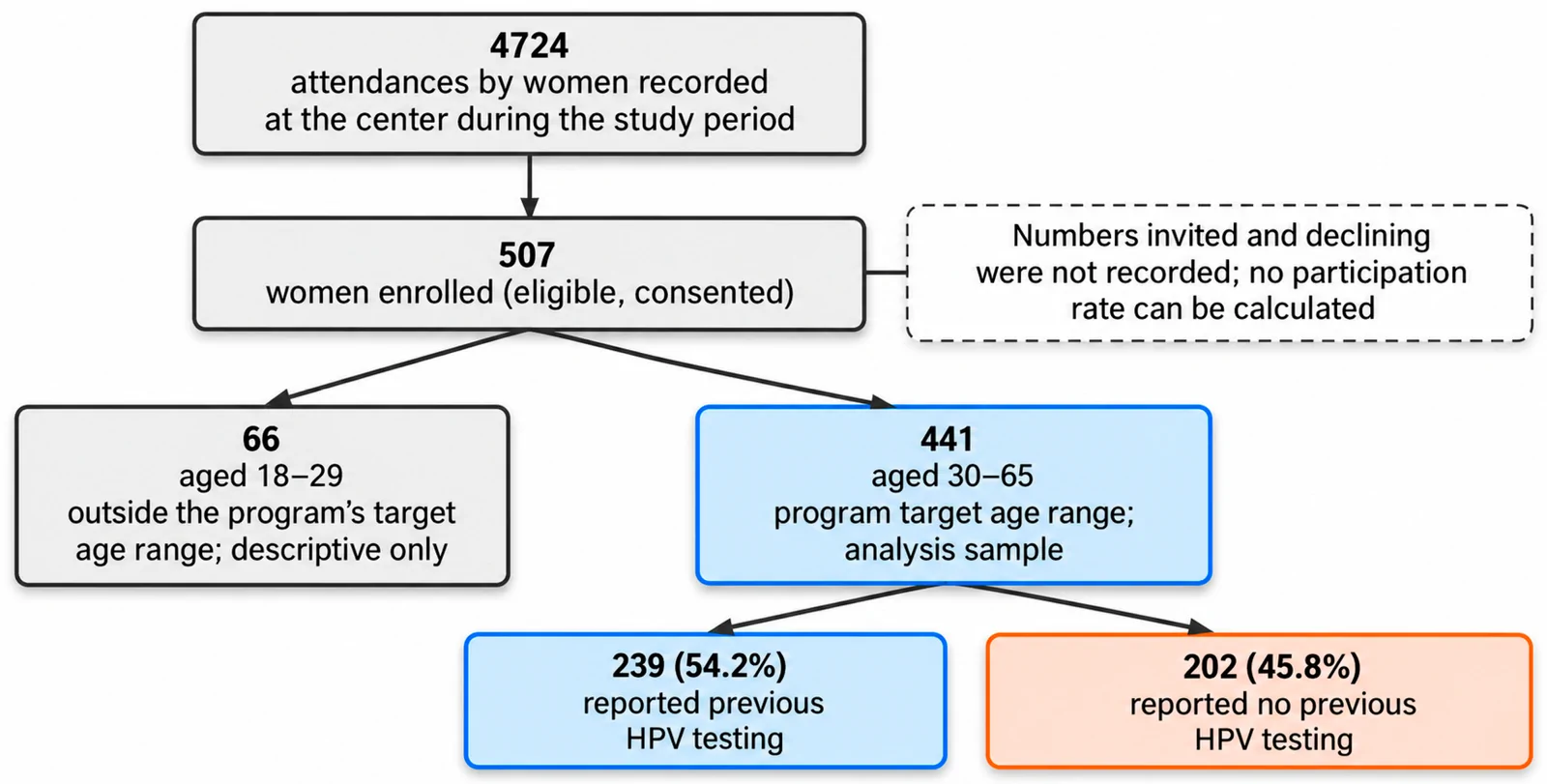
Participant flow. The center recorded attendances rather than individual women, and the numbers of women invited and of those who declined were not recorded. Therefore, a participation rate could not be calculated. Women aged 18–29 (*n* = 66) were included only in the descriptive analyses. The analysis of previous HPV testing included the 441 women aged 30–65, the target age range of the national program.

A convenience sampling method was used. Women aged 18–65 were eligible. Pregnant women and women in the first month after childbirth were excluded because screening recommendations differ for these groups. Throughout the study period, eligible women attending the center were invited to take part during their visit, and all women who agreed and gave written informed consent were interviewed. Repeat participation was not intended, and women known to have already taken part were not enrolled again. Because there was no systematic check against the consent records and the analysis dataset contains no personal identifiers, repeat participation cannot be completely ruled out. A total of 507 women were enrolled (Figure 1). Trained researchers conducted the interviews face to face and read the questions aloud to women who could not read, so that literacy was not a barrier to participation. Only women who agreed to take part were recorded. The numbers of women invited and of those who declined were not recorded, so the participation rate could not be calculated. The center recorded 4724 attendances by women during the study period, but this figure counts visits, including repeat visits by the same women, and cannot be used as a denominator. In addition, clinical eligibility for screening (such as hysterectomy status) was not verified from medical records. The sample therefore represents women in the target age range of the national screening program, whose clinical eligibility for screening was not confirmed.

Although the national program targets women aged 30–65, women aged 18–29 were also enrolled so that HPV vaccine awareness and depressive symptoms could be described in a wider adult age range. Analyses of previous HPV testing were limited to the 441 women aged 30–65.

Before data collection, the minimum sample size was calculated as 384 women, assuming an expected proportion of 50%, a 95% confidence level, a 5% margin of error and an unknown population size. Because participants were recruited by convenience sampling, this number was used as a minimum recruitment target and does not mean that the estimates have this level of precision for the wider population. Recruitment continued throughout the study period, and all 507 women who took part were included. No questionnaire was excluded. The questionnaire was piloted with 20 women attending the same center to check the clarity of the questions. Minor changes were made to the wording, and these 20 women were not included in the analysis.

The structured questionnaire covered sociodemographic characteristics, reproductive history (history of pregnancy loss or curettage, yes/no), health behaviors, awareness of HPV testing and previous HPV testing. The primary outcome was self-reported previous HPV testing, based on the question “Have you had cervical cancer screening (HPV) testing?” (yes/no; English translation). The question did not ask when, where or within which program the test was done, and the type and timing of the test were not verified from records. Therefore, a woman whose only previous test was cytology (a Pap smear) could have answered yes. Awareness of HPV testing availability was assessed at the same interview with a separate yes/no question: “Are you aware that cervical cancer screening (HPV) testing is carried out?” Depressive symptoms were assessed with the Beck Depression Inventory (BDI) [16], using the Turkish standard form [17]. The BDI total score was analyzed mainly as a continuous variable. Standard severity categories and a cut-off of ≥17 were used in the descriptive and sensitivity analyses. This cut-off was taken from the Turkish validation study, in which scores of 17 and above indicated depression above the normal range [17].

All analyses were performed in R 4.6.1. Descriptive statistics were used to summarize the sample. Bivariate comparisons were made with Pearson’s chi-square test, the independent-samples t-test or the Mann–Whitney U test, as appropriate. Crude odds ratios were estimated by univariable logistic regression. For the logistic regression models, some categories were combined: education (middle school or below vs. high school or above), marital status (single, widowed or divorced vs. married), economic status (very poor or poor vs. average, good or very good), employment (regular or temporary work vs. not employed), perceived health (very poor, poor or average vs. good or very good), and tobacco use (occasional or daily use vs. never or former use).

Multivariable logistic regression was used to examine factors associated with previous HPV testing among women in the target age range (30–65 years). Covariates were selected a priori on the basis of clinical relevance and previous studies. The BDI score was kept in both models regardless of its univariable *p*-value, because the association between depression and previous HPV testing was one of the main hypotheses of the study. Two nested models were fitted, one without (Model A) and one with (Model B) awareness of HPV testing availability, to assess how adding awareness changed the other estimates.

Model fit and multicollinearity were assessed with standard diagnostic tests. Sensitivity analyses were performed with alternative BDI categorizations, and Firth penalized regression was used because of sparse data in the awareness variable. There were no missing values in the final analysis dataset, so no imputation was needed.

Results are presented with adjusted odds ratios (aOR) and 95% confidence intervals (CI). A two-sided *p*-value < 0.05 was considered statistically significant.

The study-specific questionnaire items are provided in Supplementary File S1 in Turkish with an English translation. The copyrighted BDI items are not reproduced there.

## 3. Results

### 3.1. Participant Characteristics

A total of 507 women participated in the study. The mean age of the participants was 40.97 ± 9.79 years (median 42, IQR 35–48; range 18–65). Most of the women were married (84.6%), had at least one child (86.8%), and were not employed (74.4%). Overall, 31.8% reported at least one previous pregnancy loss or curettage. The rate of current smokers was 30.6%. The sociodemographic and clinical characteristics of the participants are presented in Table 1.

The contraceptive methods used by the participants and the reasons for not using a method are shown in Supplementary Figure S1.

In the full sample, 84.0% of participants were aware that HPV testing is carried out and 48.5% reported previous HPV testing. Awareness of the HPV vaccine was reported by 44.2% of women, while only 2.6% had received the vaccine. The mean BDI total score was 12.08 ± 11.44 (median 9, IQR 4–16; range 0–63), and the distribution was right-skewed (skewness 1.97). According to the standard severity categories, 77.9% of participants had minimal or mild symptoms, and 22.1% (*n* = 112) scored 17 or above.

Additional descriptive analyses of BDI severity by sociodemographic and clinical characteristics are provided in Supplementary Table S1.

### 3.2. Previous HPV Testing

Of all participants, 441 (87.0%) were aged 30–65, the target age range of the national program (mean age 43.5 ± 7.8 years). Of these, 239 (54.2%) reported previous HPV testing and 202 (45.8%) did not. Table 2 compares the two groups by sociodemographic, clinical and awareness characteristics.

**Figure 2:**
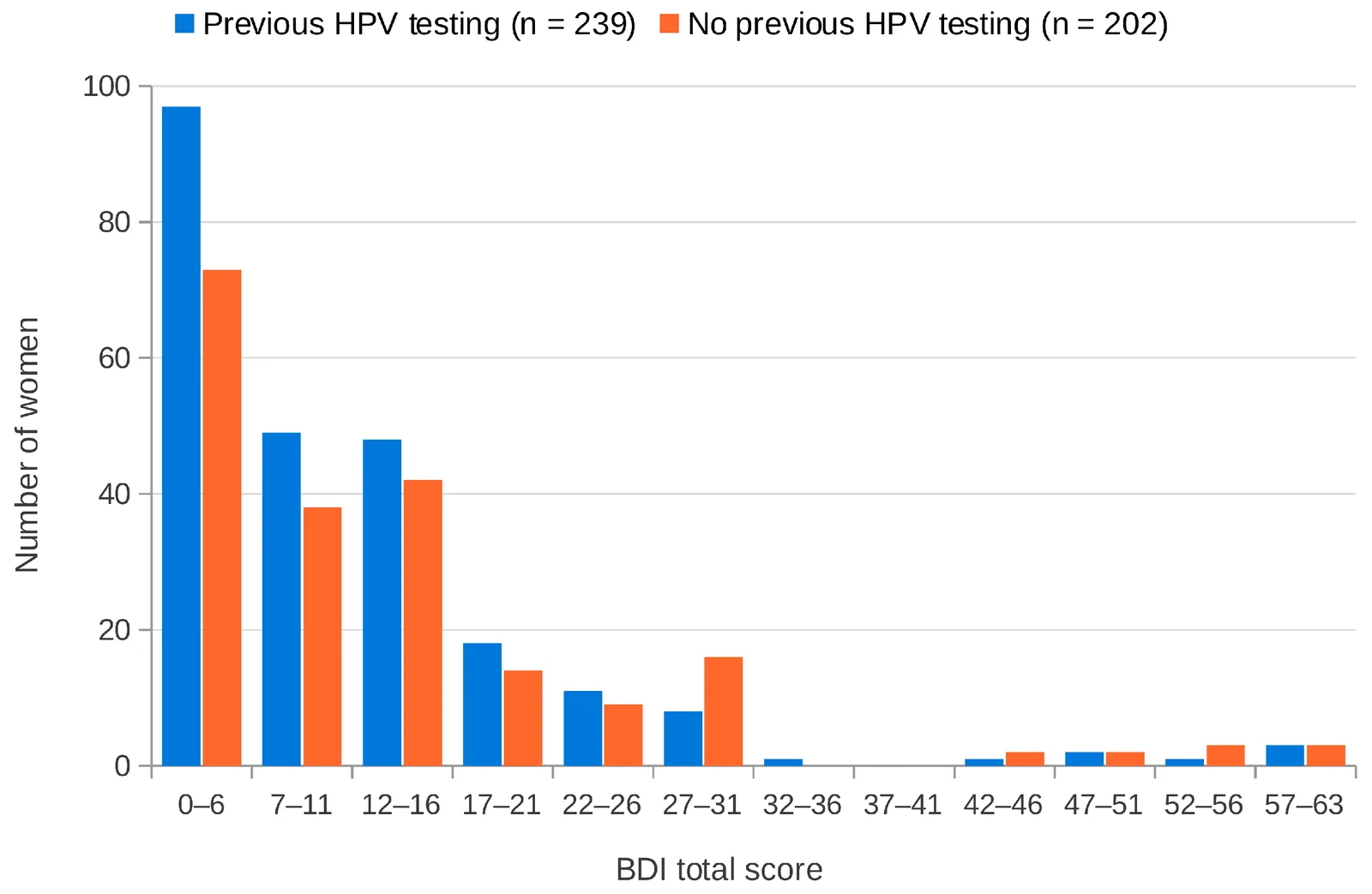
Distribution of Beck Depression Inventory (BDI) total scores among women aged 30–65 who reported previous HPV testing (*n* = 239) and those who did not (*n* = 202). Bars show the number of women in each band of the raw total score (possible range 0–63). The first and last bands are 7 points wide, and the others are 5 points wide, so that the Turkish cut-off score of 17 falls on a band boundary. Median scores were 8 (IQR 4–15) and 11 (IQR 5–16), respectively (Mann–Whitney *p* = 0.097). IQR, interquartile range.

Women who reported previous testing were older than those who did not (45.2 ± 7.5 vs. 41.4 ± 7.6 years; *p* < 0.001; crude OR 1.07 per year, 95% CI 1.04–1.10). Previous testing did not differ significantly by educational level. It was more common among married than unmarried women (55.8% vs. 39.5%; *p* = 0.042), among women with children than among those without (55.4% vs. 34.6%; *p* = 0.039), and among women reporting a previous pregnancy loss or curettage than among those reporting none (62.0% vs. 49.8%; *p* = 0.014). Women who were aware that HPV testing is carried out reported previous testing much more often than those who were not (61.6% vs. 8.2%; *p* < 0.001). No significant associations were found for HPV vaccine awareness (*p* = 0.051), HPV vaccination status (*p* = 0.095), or BDI category (*p* = 0.165). Median BDI total scores were 8 (IQR 4–15) among tested and 11 (IQR 5–16) among untested women, a difference that did not reach significance (Mann–Whitney *p* = 0.097; t-test *p* = 0.079). The crude OR for the continuous score was 0.93 per 5-point increase (95% CI 0.85–1.01; *p* = 0.081), and no clear association was found after adjustment (Section 3.3 and Section 3.4). The distribution of raw BDI totals in the two groups is shown in Figure 2.

### 3.3. Multivariable Analysis

The results of the two nested multivariable logistic regression models are shown in Table 3. In Model A, which did not include awareness, increasing age (aOR = 1.071 per year; 95% CI: 1.041–1.102; *p* < 0.001) and a history of pregnancy loss or curettage (aOR = 1.540; 95% CI: 1.011–2.347; *p* = 0.044) were independently associated with previous testing. Both associations remained after awareness of HPV testing availability was added in Model B (age aOR = 1.073; 95% CI: 1.040–1.107; *p* < 0.001; pregnancy loss or curettage aOR = 1.621; 95% CI: 1.024–2.567; *p* = 0.039). Awareness showed the strongest association (aOR = 21.349; 95% CI: 8.066–56.509; *p* < 0.001). The BDI total score was not independently associated with previous testing in either model (Model A aOR = 0.989 per point, 95% CI 0.970–1.008, *p* = 0.260; Model B aOR = 0.991, 95% CI 0.970–1.011, *p* = 0.365).

Adding awareness improved model fit (likelihood ratio χ^2^ = 67.13, df = 1, *p* < 0.001) and discrimination (C-statistic 0.670 vs. 0.765; Nagelkerke R^2^ 0.122 vs. 0.294). The adjusted odds ratios for age, marital status, having children, pregnancy loss or curettage, employment, perceived economic status and BDI score changed by less than 7%. Larger changes were observed for education (−19.1%), smoking (−15.4%) and perceived health status (−14.6%), although none of these three variables was statistically significant in either model. There was no evidence of multicollinearity in either model (all VIF ≤ 1.23; all tolerance values ≥ 0.81). The Hosmer–Lemeshow test did not indicate lack of fit in either model (*p* = 0.155 for Model A and *p* = 0.633 for Model B).

### 3.4. Sensitivity Analyses

Depressive symptoms. No clear association between depressive symptoms and previous testing was found in any of the analyses (Table 4). Adding a quadratic term showed no evidence of departure from linearity on the log-odds scale (*p* = 0.403). The point estimates decreased across the four severity categories (0.707, 0.696 and 0.604 compared with minimal depression), but all confidence intervals were wide and included 1. Therefore, these data do not show whether there is a graded relationship.

Awareness of HPV testing availability. Only 5 of the 61 women who were not aware of HPV testing had been tested. Profile-likelihood confidence intervals gave a wider upper limit than the Wald intervals, but the result did not change (aOR = 21.35; 95% CI: 8.82–64.16). Firth penalized logistic regression, which reduces small-sample and sparse-data bias, gave a slightly smaller but still very large estimate (aOR = 18.42; 95% CI: 7.89–52.29; *p* < 0.001).

## 4. Discussion

In this cross-sectional study of women attending a Healthy Life Center in Istanbul, 54.2% of women in the target age range of the national screening program reported that they had ever had an HPV test. This figure is a self-reported proportion from a single clinic. It cannot be compared with national participation estimates [8] or with the WHO target of 70% screening coverage [2], which is defined at the population level and for specific ages. Because the questionnaire did not record when or within which program the test was done, we also cannot judge whether this level of testing is high or low in relation to the aims of the program.

The strongest association in the adjusted model was between awareness of HPV testing availability and previous testing (aOR = 21.35). The association does not show that awareness leads to testing. Both were reported at the same interview, so we do not know which came first. Women who have been tested are likely to know about the program because they have used it, and the awareness question could not separate knowledge gained before a test from knowledge gained through it. Reverse causality and recall bias may therefore explain at least part of this association. Whether raising awareness would increase participation should be tested in prospective or interventional studies. A Cochrane review of 70 randomized trials (69 contributing to the meta-analysis; 257,899 participants) found moderate-certainty evidence that invitation letters increase cervical screening uptake and low-certainty evidence for educational materials, and the most effective format was unclear [18]. However, these trials were conducted in other settings and tested invitation and education programs, whereas our study measured awareness.

The association remained strong in the Firth penalized regression (aOR = 18.42). Firth estimation corrects for sparse-data bias, but it does not address reverse causality, common-method bias or misclassification of the self-reported variables. The confidence interval was also wide. An estimate of this size from observational data indicates a strong association, but its exact size should be interpreted with caution. The lack of a significant relationship between HPV vaccine awareness, vaccination status and previous testing may be related to the low prevalence of HPV vaccination in this age group (2.6% overall) and to the differences in the target groups for screening and vaccination [2].

The BDI total score was not clearly associated with previous testing in either model. The results were similar in the sensitivity analyses, in which the score was used on its original 0–63 scale, dichotomized at the validated cut-off of 17, grouped into four severity categories or divided into quartiles. There was no evidence of a graded relationship or of departure from linearity. However, the confidence intervals do not exclude a modest association, so our results should not be taken as evidence that depressive symptoms are unrelated to HPV testing. Reviews have reported lower participation in some cancer screening programs among people with depression, but the findings vary between programs and are inconclusive for cervical screening [13,19]. Proposed mechanisms, such as reduced self-care or low motivation, were not measured in our study.

Two aspects of the study design should be considered when interpreting this finding. First, the BDI measures symptoms during the past week, whereas the HPV test may have been done much earlier. Second, all participants were already attending a primary care facility. If depressive symptoms prevent some women from reaching health services, this would not be seen in a sample recruited in a health facility. We also did not collect data on how services were organized, so we could not examine whether this affects the relationship between depressive symptoms and HPV testing.

Older age was associated with previous testing after adjustment, which is consistent with national survey findings [8]. Older women may have more contact with health services, for example, for the follow-up of chronic diseases, and they have been eligible for screening for a longer period. A history of pregnancy loss or curettage was also associated with previous testing. This finding should be interpreted with caution because the confidence interval was wide and close to 1, several associations were examined without adjustment for multiple comparisons, and we found no directly comparable published studies. We therefore regard it as exploratory, and it needs to be replicated. US population survey data show that having a usual source of care and insurance coverage are associated with higher screening test receipt [20], although these data concern continuity of care and not the effect of a single visit.

No significant association was found between previous testing and education level, perceived economic status or employment status. Cost is one possible explanation. Services at Healthy Life Centers are provided free of charge [15], and HPV screening is provided free of charge to eligible women through Türkiye’s primary care screening program [7,21]. Free services may reduce the financial barriers that lead to socioeconomic differences in screening in other settings. However, this is a hypothesis, and our design cannot show that the national program has reduced health inequalities. In addition, our sample came from a single center in one district of Istanbul and was fairly homogeneous in socioeconomic terms, with only 6.8% of women reporting a poor or very poor economic situation. The lack of a socioeconomic gradient may therefore reflect limited variation and limited statistical power.

### Strengths and Limitations

The strengths of this study include a sample of 507 women, the use of the validated Beck Depression Inventory and the restriction of the main analysis to women in the target age range of the national screening program. The study also has several limitations. First, the cross-sectional design does not allow causal inference, including for the association between awareness and testing. Second, convenience sampling was used at a single center, and all participants were already using health services. The findings therefore cannot be generalized to all women in Türkiye. Because the numbers of women invited and of those who declined were not recorded, the participation rate is unknown, and selection bias cannot be quantified. Repeat participation by the same woman also cannot be completely ruled out.

Third, the data were self-reported and were not verified against registry records, so recall bias and misclassification are possible. Because the timing and type of the previous test were not recorded or verified, the outcome reflects ever-testing and does not show whether women were up to date with screening. It may also include women whose only previous test was cytology (a Pap smear). Fourth, awareness was measured with a single yes/no question, which cannot capture differences in health literacy. Fifth, the BDI cut-off of 17 comes from a validation study in university students.

In addition, miscarriages and curettages were combined into a single variable, so spontaneous pregnancy loss could not be examined separately. We did not adjust for multiple comparisons, and findings of borderline significance should be regarded as exploratory. Finally, only five women who had been tested were unaware of HPV testing, so the estimate for awareness was imprecise and had a wide confidence interval.

## 5. Conclusions

Among 441 women aged 30–65 attending a Healthy Life Center in Istanbul, 239 (54.2%) reported having had an HPV test at least once. This clinic-based, self-reported figure does not reflect population screening coverage. Awareness of HPV testing availability was the factor most strongly associated with previous testing, but because both were reported at the same interview, the study cannot show which came first or whether awareness-raising would increase testing. No clear association was found with depressive symptoms, although the estimates were imprecise.

As a practical implication, and not as a finding of this study, primary care visits reach many women in the program’s target age range and may be a useful opportunity to discuss cervical cancer screening. Prospective or interventional studies are needed to test whether raising awareness in this setting increases participation, ideally with previous testing verified from program records instead of self-report.

## Figures and Tables

**Table 1:** Sociodemographic and clinical characteristics of participants (N = 507).

Variable	Category	*n*	%
Marital status	Single	50	9.9
	Married	429	84.6
	Widowed/Divorced	28	5.5
Has children	No	67	13.2
	Yes	440	86.8
History of pregnancy loss or curettage	No	346	68.2
	Yes	161	31.8
Education	Illiterate	1	0.2
	Literate	18	3.6
	Primary school	178	35.1
	Middle school	71	14.0
	High school	155	30.6
	University or above	84	16.6
Employment status	Regular employment	109	21.5
	Temporary employment	21	4.1
	Unemployed	377	74.4
Perceived economic status	Very poor	6	1.2
	Poor	29	5.7
	Average	357	70.4
	Good	110	21.7
	Very good	5	1.0
Perceived health status	Very poor	4	0.8
	Poor	18	3.6
	Average	243	47.9
	Good	215	42.4
	Very good	27	5.3
Tobacco use	Never	312	61.5
	Occasional	62	12.2
	Daily smoker	93	18.3
	Former smoker	40	7.9

Values are numbers of women and percentages of all 507 participants.

**Table 2:** Self-reported previous HPV testing by sociodemographic, clinical and awareness characteristics among women aged 30–65 (*n* = 441).

Variable	Category	Tested, *n* (%)	Not Tested, *n* (%)	Crude OR (95% CI)	*p*
Education	≤Middle school	147 (57.2)	110 (42.8)	1 (ref)	0.135
	≥High school	92 (50.0)	92 (50.0)	0.75 (0.51–1.09)	
Marital status	Single/widowed/divorced	17 (39.5)	26 (60.5)	1 (ref)	0.042
	Married	222 (55.8)	176 (44.2)	1.93 (1.02–3.67)	
Has children	No	9 (34.6)	17 (65.4)	1 (ref)	0.039
	Yes	230 (55.4)	185 (44.6)	2.35 (1.02–5.39)	
Perceived economic status	Poor	16 (53.3)	14 (46.7)	1 (ref)	0.922
	Average–good	223 (54.3)	188 (45.7)	1.04 (0.49–2.18)	
Employment status	Employed	66 (56.9)	50 (43.1)	1.16 (0.76–1.78)	0.496
	Not employed	173 (53.2)	152 (46.8)	1 (ref)	
Perceived health status	Poor–average	130 (53.5)	113 (46.5)	1 (ref)	0.745
	Good–very good	109 (55.1)	89 (44.9)	1.07 (0.73–1.55)	
Tobacco use	Never/former	170 (55.7)	135 (44.3)	1 (ref)	0.330
	Current smoker	69 (50.7)	67 (49.3)	0.82 (0.55–1.23)	
History of pregnancy loss or curettage	No	141 (49.8)	142 (50.2)	1 (ref)	0.014
	Yes	98 (62.0)	60 (38.0)	1.65 (1.11–2.45)	
Awareness of HPV testing availability	No	5 (8.2)	56 (91.8)	1 (ref)	<0.001
	Yes	234 (61.6)	146 (38.4)	17.95 (7.03–45.86)	
HPV vaccine awareness	No	127 (50.2)	126 (49.8)	1 (ref)	0.051
	Yes	112 (59.6)	76 (40.4)	1.46 (1.00–2.14)	
HPV vaccine received	No	229 (53.5)	199 (46.5)	1 (ref)	0.095
	Yes	10 (76.9)	3 (23.1)	2.90 (0.79–10.67)	
BDI total score ≥ 17	No (0–16)	194 (55.9)	153 (44.1)	1 (ref)	0.165
	Yes (17–63)	45 (47.9)	49 (52.1)	0.72 (0.46–1.14)	
BDI total score, continuous	per 5-point increase	—	—	0.93 (0.85–1.01)	0.081 ^a^
Total		239 (54.2)	202 (45.8)		

Percentages are row percentages. Crude odds ratios (OR) with 95% confidence intervals (CI) are from univariable logistic regression; ref, reference category. *p* values for categorical variables are from Pearson’s chi-square test; the smallest expected cell count was 5.95, so no exact test was required. BDI, Beck Depression Inventory; HPV, human papillomavirus. ^a^ Wald test from univariable logistic regression of the continuous BDI total score.

**Table 3:** Multivariable logistic regression models of self-reported previous HPV testing among women aged 30–65 (*n* = 441).

Variable	Comparison (Reference)	Model A aOR (95% CI)	*p*	Model B aOR (95% CI)	*p*
Age	per 1-year increase	1.071 (1.041–1.102)	<0.001	1.073 (1.040–1.107)	<0.001
Marital status	married (single/widowed/divorced)	1.521 (0.725–3.191)	0.267	1.451 (0.659–3.195)	0.355
Education	≥high school (<high school)	0.994 (0.651–1.518)	0.978	0.805 (0.510–1.269)	0.350
Employment status	employed (not employed)	1.378 (0.869–2.185)	0.172	1.445 (0.879–2.376)	0.146
Perceived economic status	average–good (poor)	0.951 (0.421–2.150)	0.904	1.017 (0.418–2.471)	0.971
Has children	yes (no)	2.042 (0.816–5.112)	0.127	1.947 (0.741–5.113)	0.176
History of pregnancy loss or curettage	yes (no)	1.540 (1.011–2.347)	0.044	1.621 (1.024–2.567)	0.039
Tobacco use	current smoker (never/former)	0.907 (0.589–1.398)	0.659	0.768 (0.484–1.219)	0.262
Perceived health status	good–very good (poor–average)	1.135 (0.742–1.735)	0.560	0.969 (0.611–1.538)	0.895
BDI total score	per 1-point increase	0.989 (0.970–1.008)	0.260	0.991 (0.970–1.011)	0.365
Awareness of HPV testing availability	yes (no)	not included	—	21.349 (8.066–56.509)	<0.001
Model fit					
C-statistic (AUC)		0.670		0.765	
Nagelkerke R^2^		0.122		0.294	
Hosmer–Lemeshow		χ^2^ = 11.93, df = 8	0.155	χ^2^ = 6.12, df = 8	0.633
Maximum VIF		1.22		1.23	

Outcome: self-reported previous HPV testing (239 of 441 women). Reference categories are given in parentheses. Model A: demographic, clinical and depression covariates. Model B: Model A plus awareness of HPV testing availability. The BDI enters both models as the raw total score (0–63). *p*-values are from Wald tests, and confidence intervals are Wald intervals. Likelihood ratio test for the addition of awareness: χ^2^ = 67.13, df = 1, *p* < 0.001. aOR, adjusted odds ratio; CI, confidence interval; BDI, Beck Depression Inventory; AUC, area under the receiver operating characteristic curve; VIF, variance inflation factor.

**Table 4:** Sensitivity analyses for the association between depressive symptoms and self-reported previous HPV testing (*n* = 441).

BDI Parameterization	Comparison	aOR (95% CI)	*p*	AIC
Raw total score (primary analysis)	per 1-point increase	0.991 (0.970–1.011)	0.365	522.87
	per 5-point increase	0.953 (0.860–1.057)	0.365	
Dichotomy at the validated cut-off	≥17 vs. 0–16	0.786 (0.446–1.385)	0.406	523.00
Four severity categories	mild (10–16) vs. minimal (0–9)	0.707 (0.417–1.199)	0.198	525.27
	moderate (17–29) vs. minimal	0.696 (0.356–1.362)	0.290	
	severe (30–63) vs. minimal	0.604 (0.212–1.718)	0.345	
Quartiles of the total score	Q2 (5–9) vs. Q1 (0–4)	1.055 (0.579–1.923)	0.861	525.31
	Q3 (10–16) vs. Q1	0.726 (0.397–1.328)	0.298	
	Q4 (17–63) vs. Q1	0.691 (0.349–1.369)	0.289	

Outcome: self-reported previous HPV testing (239 of 441 women). All models are adjusted for the Model B covariate set (Table 3); the first row repeats the Model B estimate for comparison. *p*-values are from Wald tests. Test for departure from linearity (quadratic term): *p* = 0.403. aOR, adjusted odds ratio; CI, confidence interval; AIC, Akaike information criterion (lower values indicate better fit).

## Data Availability

The data supporting the findings of this study are available upon reasonable request but are subject to approval by the Health Directorate of Istanbul.

## Supplementary Materials

The following supporting information can be downloaded at: https://www.mdpi.com/article/10.3390/healthcare14203428/s1, Figure S1: Contraceptive methods used and reasons for non-use (N = 507); Table S1: Depressive symptom severity by sociodemographic and clinical characteristics (N = 507); File S1: Study questionnaire, Turkish original with English translation.

## Abbreviations

The following abbreviations are used in this manuscript:

AIC	Akaike information criterion
aOR	Adjusted odds ratio
AUC	Area under the receiver operating characteristic curve
BDI	Beck Depression Inventory
CI	Confidence interval
HPV	Human papillomavirus
IQR	Interquartile range
OR	Odds ratio
STROBE	Strengthening the Reporting of Observational Studies in Epidemiology
VIF	Variance inflation factor
WHO	World Health Organization

## References

[1] Singh D., Vignat J., Lorenzoni V., Eslahi M., Ginsburg O., Lauby-Secretan B., Arbyn M., Basu P., Bray F., Vaccarella S. (2023). Global estimates of incidence and mortality of cervical cancer in 2020: A baseline analysis of the WHO Global Cervical Cancer Elimination Initiative. Lancet Glob. Health.

[2] World Health Organization Cervical Cancer. https://www.who.int/news-room/fact-sheets/detail/cervical-cancer.

[3] Centers for Disease Control and Prevention Cancers Associated with Human Papillomavirus. U.S. Cancer Statistics. https://www.cdc.gov/united-states-cancer-statistics/publications/hpv-associated-cancers.html.

[4] Koliopoulos G., Nyaga V.N., Santesso N., Bryant A., Martin-Hirsch P.P.L., Mustafa R.A., Schünemann H., Paraskevaidis E., Arbyn M. (2017). Cytology versus HPV testing for cervical cancer screening in the general population. Cochrane Database Syst. Rev..

[5] European Commission, Joint Research Centre (2025). European Guidelines on Cervical Cancer Screening and Diagnosis: Screening Populations Aged 30 to 50 Years. https://cancer-screening-and-care.jrc.ec.europa.eu/en/ec-cvc/european-cervical-cancer-guidelines?topic=328&usertype=327.

[6] T.C. Sağlık Bakanlığı Halk Sağlığı Genel Müdürlüğü Kanser Taramaları [Cancer Screenings]. https://hsgm.saglik.gov.tr/tr/kanser-taramalari.

[7] Arslan H.N., Oruc M.A. (2022). Results from a cervical cancer screening program in Samsun, Turkey. BMC Women’s Health.

[8] Gülle B.T., Tozduman B., Ören Çelik M.M. (2025). Determinants of cancer screening participation in Türkiye: A nationwide study of demographic, socioeconomic, and lifestyle factors. BMC Public Health.

[9] Oldach B.R., Katz M.L. (2014). Health literacy and cancer screening: A systematic review. Patient Educ. Couns..

[10] Chorley A.J., Marlow L.A.V., Forster A.S., Haddrell J.B., Waller J. (2017). Experiences of cervical screening and barriers to participation in the context of an organised programme: A systematic review and thematic synthesis. Psycho-Oncol..

[11] Kim H.W., Kim D.H. (2015). Awareness of cervical cancer prevention among mothers of adolescent daughters in Korea: Qualitative research. BMJ Open.

[12] Daniati N., Widjaja G., Olalla Gracìa M., Chaudhary P., Nader Shalaby M., Chupradit S., Fakri Mustafa Y. (2021). The Health Belief Model’s application in the development of health behaviors. Health Educ. Health Promot..

[13] Mkuu R., Salloum R.G., Shenkman E., Schaefer N., Le T., Jorratt A., Meduri Y., Goede D., Lee J.-H., Staras S.A.S. (2023). Screening for cervical cancer among women with behavioral health conditions—A systematic review. Prev. Med. Rep..

[14] US Preventive Services Task Force (2023). Screening for depression and suicide risk in adults: US Preventive Services Task Force recommendation statement. JAMA.

[15] T.C. Sağlık Bakanlığı Halk Sağlığı Genel Müdürlüğü. Sağlıklı Hayat Merkezleri [Healthy Life Centers]. https://hsgm.saglik.gov.tr/tr/shm.

[16] Beck A.T., Ward C.H., Mendelson M., Mock J., Erbaugh J. (1961). An inventory for measuring depression. Arch. Gen. Psychiatry.

[17] Hisli N. (1989). Beck depresyon envanterinin üniversite öğrencileri için geçerliği, güvenirliği [A reliability and validity study of the Beck Depression Inventory in a university student sample]. Psikol. Derg..

[18] Staley H., Shiraz A., Shreeve N., Bryant A., Martin-Hirsch P.P.L., Gajjar K. (2021). Interventions targeted at women to encourage the uptake of cervical screening. Cochrane Database Syst. Rev..

[19] Young B., Robb K.A. (2021). Understanding patient factors to increase uptake of cancer screening: A review. Future Oncol..

[20] Sabatino S.A., Thompson T.D., White M.C., Shapiro J.A., de Moor J., Doria-Rose V.P., Clarke T., Richardson L.C. (2021). Cancer screening test receipt—United States, 2018. MMWR Morb. Mortal. Wkly. Rep..

[21] Gultekin M., Zayifoglu Karaca M., Kucukyildiz I., Dundar S., Boztas G., Turan H.S., Hacikamiloglu E., Murtuza K., Keskinkilic B., Sencan I. (2018). Initial results of population based cervical cancer screening program using HPV testing in one million Turkish women. Int. J. Cancer.

