# Supplementary material for: Factors Associated with Self-Reported Previous HPV Testing Among Women Attending a Primary Healthcare Facility in Istanbul, Türkiye: A Cross-Sectional Study
Source: Healthcare (Basel). 2026 Oct 14;14(20):3428. doi: 10.3390/healthcare14203428 (PMC13704237; doi:10.3390/healthcare14203428)
Supplement: Supplementary file 1 [file healthcare-14-03428-s001.zip › Figure S1.pdf]

Figure S1. Contraceptive methods used and reasons for non-use (N = 507)

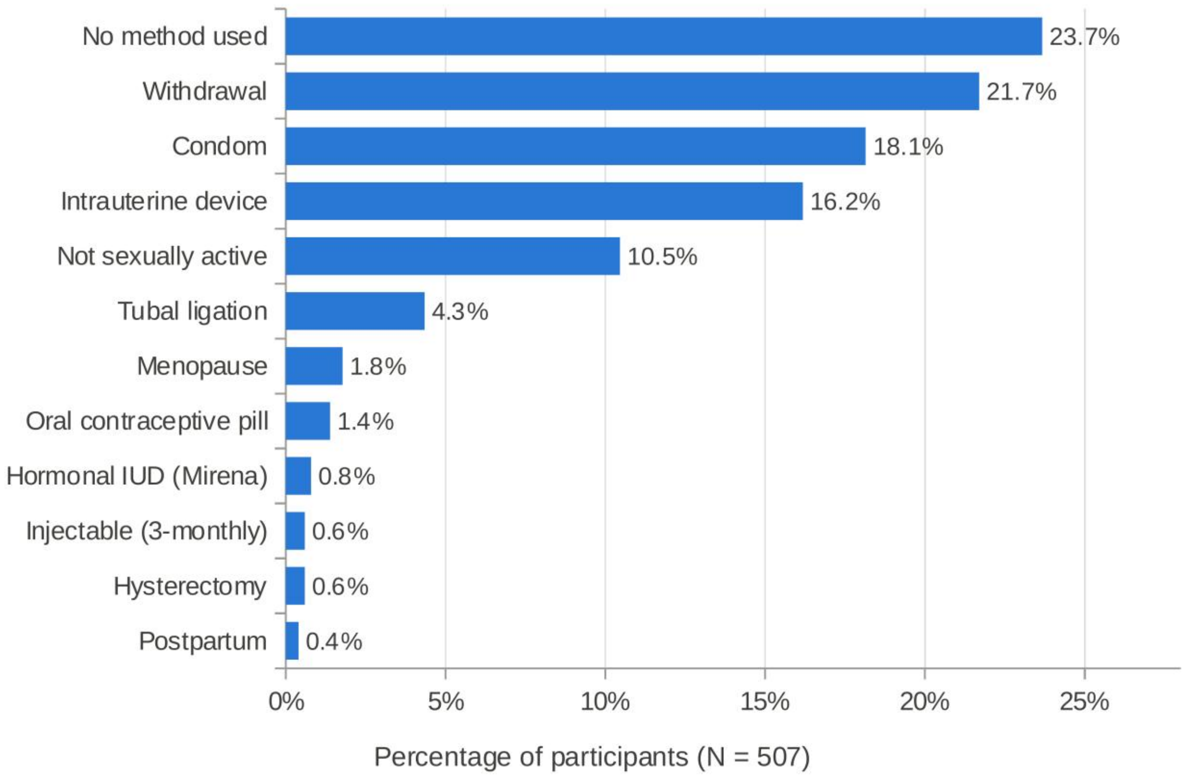

Percentages are based on all 507 participants. Menopause, hysterectomy, postpartum status and not being sexually active are reasons for not using a method. The questionnaire option for the intrauterine device did not specify the type of device, and the hormonal IUD (Mirena) was a separate option. No participant reported using a monthly injectable. The question is item 10 in Supplementary File S1. IUD, intrauterine device.
