# Supplementary material for: Factors Associated with Self-Reported Previous HPV Testing Among Women Attending a Primary Healthcare Facility in Istanbul, Türkiye: A Cross-Sectional Study
Source: Healthcare (Basel). 2026 Oct 14;14(20):3428. doi: 10.3390/healthcare14203428 (PMC13704237; doi:10.3390/healthcare14203428)
Supplement: Supplementary file 1 [file healthcare-14-03428-s001.zip › File S1.pdf]

File S1. Study questionnaire (Turkish original with English translation)

The questionnaire included 15 study-specific questions, which were asked by the researchers in face-to-face interviews. The Turkish column shows the questions as they were asked, and the English column gives their translation. The last section of the questionnaire was the Beck Depression Inventory. Because it is a copyrighted instrument, its items are not reproduced here (see the note at the end of this file).

#	Turkish original	English translation	Response options
1	Yaşınız	Your age	Open (years)
2	Medeni durumunuz	Marital status	1 Bekar (single); 2 Evli (married); 3 Dul/boşanmış/ayrı (widowed/divorced/separated)
3	Çocuğunuz var mı?	Do you have children?	1 Evet, kaç tane belirtiniz (yes, state how many); 2 Hayır (no)
4	Varsa düşük/küretaj sayınız	Number of miscarriages/curettages, if any	Open (count)
5	Öğrenim durumunuz nedir?	What is your educational attainment?	1 Okur-yazar değil (illiterate); 2 Okur-yazar (literate, no formal schooling); 3 İlköğretim (primary); 4 Ortaokul (middle school); 5 Lise (high school); 6 Üniversite ve üstü (university or above)
6	Halen gelir getiren bir işte çalışıyor musunuz?	Are you currently in paid employment?	1 Evet, düzenli işim var (yes, regular job); 2 Evet, geçici işlerde çalışıyorum (yes, temporary work); 3 Hayır, çalışmıyorum (no)
7	Ekonomik durumunuzu nasıl değerlendirirsiniz?	How do you rate your economic situation?	1 Çok kötü (very poor); 2 Kötü (poor); 3 Orta (average); 4 İyi (good); 5 Çok iyi (very good)
8	Genel olarak sağlığınızı nasıl değerlendirirsiniz?	How do you rate your health in general?	1 Çok kötü (very poor); 2 Kötü (poor); 3 Orta (average); 4 İyi (good); 5 Çok iyi (very good)

9	Tütün ürünü (sigara, elektronik sigara, nargile vb.) kullanıyor musunuz?	Do you use tobacco products (cigarettes, e-cigarettes, waterpipe, etc.)?	1 Hiç içmedim (never smoked); 2 Ara sıra içiyorum (occasional); 3 Sürekli düzenli içiyorum, günde en az 1 adet (daily, at least one per day); 4 Eskiden içiyordum, bıraktım (former smoker)
10	Kullandığınız aile planlaması yöntemi	Contraceptive method used	1 Rahim içi araç, spiral (intrauterine device); 2 Kondom, prezervatif (condom); 3 Hap, mini hap (oral contraceptive pill); 4 Geri çekme (withdrawal); 5 İğne, aylık (monthly injectable); 6 İğne, 3 aylık (3-monthly injectable); 7 Tüp ligasyonu (tubal ligation); 8 Yöntem kullanmıyorum (no method used); 9 Menopoz (menopause); 10 İlişki yok (not sexually active); 11 Lohusa, … günlük (postpartum, … days); 12 Histerektomi (hysterectomy); 13 Mirena (hormonal intrauterine device)
11	Kullandığınız doğum kontrol yöntemine kim karar verdi?	Who decided on the contraceptive method you use?	1 Ben karar verdim (I decided); 2 Eşim karar verdi (my husband decided); 3 Ortak karar verdik (joint decision)
12	Rahim ağzı kanser tarama (HPV) testi yapıldığını biliyor musunuz?	Are you aware that cervical cancer screening (HPV) testing is carried out?	1 Evet (yes); 2 Hayır (no)
13	Rahim ağzı kanser tarama (HPV) testi yaptırdınız mı?	Have you had cervical cancer screening (HPV) testing?	1 Evet yaptırdım (yes); 2 Hayır yaptırmadım (no)
14	Rahim ağzı kanserinden korunmak için HPV aşısı olduğunu biliyor musunuz?	Are you aware that an HPV vaccine exists for protection against cervical cancer?	1 Evet (yes); 2 Hayır (no)
15	Rahim ağzı kanserinden	Have you received the HPV vaccine for	1 Evet (yes); 2 Hayır (no)

	korunmak için HPV aşısı yaptırdınız mı?	protection against cervical cancer?	

Notes on individual items

Item 4 (reproductive history). The question asked for the total number of miscarriages and curettages (*düşük/küretaj*). In Turkish clinical usage, *küretaj* (curettage) refers both to the surgical management of a miscarriage and to induced abortion. Because the question combined these events and no separate question on induced abortion was asked, the variable is reported in the manuscript as a history of pregnancy loss or curettage. It was analyzed as a binary variable (one or more events vs. none).

Item 10 (contraceptive method). Four of the 13 response options (menopause, hysterectomy, postpartum status and not being sexually active) are reasons for not using a method. For this reason, Figure S1 is titled “Contraceptive methods used and reasons for non-use”. For the postpartum option, the number of days since delivery was recorded as free text. This information was used during recruitment to apply the postpartum exclusion criterion and was not entered into the analysis dataset.

Item 11 (decision on the contraceptive method). The question referred to the method in use, but it was answered by all participants, including women who did not use a method. Because its meaning is unclear for these women, this item was not used in the analyses.

Items 12 and 13 (awareness and previous HPV testing). Both are yes/no questions and were asked at the same interview. Item 12 shows whether a woman knew that the test is carried out; it does not measure knowledge of the eligibility criteria, the five-year interval or the fact that the test is free. Item 13 does not record when, where or within which program the test was done, and it does not distinguish HPV DNA testing from cytology. The outcome therefore reflects ever-testing and may include women whose only previous test was cytology.

Beck Depression Inventory

The last section of the questionnaire was the Beck Depression Inventory. We used the published Turkish standard form validated by Hisli (reference 17 of the manuscript) without any changes to the items, response options or scoring. Because the inventory is copyrighted, its 21 items are not reproduced here. The original inventory was developed by Beck et al. (reference 16). Following the standard instructions, respondents chose, for each group of statements, the one that best described how they had felt during the past week, including the day of the interview. Each item is scored 0–3, and the total score ranges from 0 to 63.
