# Supplementary material for: Factors Associated with Self-Reported Previous HPV Testing Among Women Attending a Primary Healthcare Facility in Istanbul, Türkiye: A Cross-Sectional Study
Source: Healthcare (Basel). 2026 Oct 14;14(20):3428. doi: 10.3390/healthcare14203428 (PMC13704237; doi:10.3390/healthcare14203428)
Supplement: Supplementary file 1 [file healthcare-14-03428-s001.zip › Table S1.pdf]

Table S1. Depressive symptom severity by sociodemographic and clinical characteristics (N = 507)

Variable	Category	Minimal–mild, n (%)	Moderate–severe, n (%)	p
Age group	18–40	172 (75.4)	56 (24.6)	0.225
	41–65	223 (79.9)	56 (20.1)	
Marital status	Single/widowed/divorced	50 (64.1)	28 (35.9)	**0.001**
	Married	345 (80.4)	84 (19.6)	
Education	≤ Middle school	210 (78.4)	58 (21.6)	0.796
	≥ High school	185 (77.4)	54 (22.6)	
Has children	No	52 (77.6)	15 (22.4)	0.950
	Yes	343 (78.0)	97 (22.0)	
Perceived economic status	Poor	21 (60.0)	14 (40.0)	**0.008**
	Average–good	374 (79.2)	98 (20.8)	
Employment status	Employed	104 (80.0)	26 (20.0)	0.505
	Not employed	291 (77.2)	86 (22.8)	
Perceived health status	Poor–average	178 (67.2)	87 (32.8)	**<0.001**
	Good–very good	217 (89.7)	25 (10.3)	
Tobacco use	Never/former	291 (82.7)	61 (17.3)	**<0.001**
	Current smoker	104 (67.1)	51 (32.9)	
History of pregnancy loss or curettage	No	283 (81.8)	63 (18.2)	**0.002**
	Yes	112 (69.6)	49 (30.4)	
**Total**		**395 (77.9)**	**112 (22.1)**	

Depressive symptom severity was assessed with the Beck Depression Inventory and dichotomized as minimal–mild (0–16) versus moderate–severe (17–63). p values are from Pearson's chi-square test. The smallest expected cell count was 7.73, so no exact test was required. Percentages are row percentages.

Moderate–severe depressive symptoms were more frequent among unmarried women (35.9% vs. 19.6%; $p = 0.001$), women reporting a poor economic situation (40.0% vs. 20.8%; $p = 0.008$), women reporting poor-to-average health (32.8% vs. 10.3%; $p < 0.001$), current smokers (32.9% vs. 17.3%; $p < 0.001$) and women reporting a previous pregnancy loss or curettage (30.4% vs. 18.2%; $p = 0.002$). No associations were observed with age group, education, having children or employment status.
